# Mechanisms of oestrogen receptor (ER) gene regulation in breast cancer

**DOI:** 10.1530/EJE-16-0124

**Published:** 2016-07

**Authors:** J S Carroll

**Affiliations:** Cancer Research UKCambridge Institute, University of Cambridge, Cambridge, UK

## Abstract

Most breast cancers are driven by a transcription factor called oestrogen receptor (ER). Understanding the mechanisms of ER activity in breast cancer has been a major research interest and recent genomic advances have revealed extraordinary insights into how ER mediates gene transcription and what occurs during endocrine resistance. This review discusses our current understanding on ER activity, with an emphasis on several evolving, but important areas of ER biology.

## Invited Author’s profile


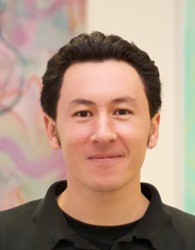


**Dr Jason Carroll** was born in Australia and completed his PhD degree in Sydney at the Garvan Institute. He then did postdoctoral work with Prof Myles Brown at Dana-Farber Cancer Institute at Harvard Medical School. Dr Carroll joined Cancer Research UK, University of Cambridge as a group leader in 2006 and is currently a senior group leader and a Fellow at Clare College, University of Cambridge. His main research interest is in understanding how oestrogen receptor (ER) regulates gene expression in breast cancer and recently has been focussing on delineating the hormonal cross-talk that exists between ER and other hormonal nuclear receptor pathways.

## Introduction

Oestrogen receptor (ER) is a transcription factor that regulates gene expression events that culminate in cell division, an important property that contributes to its critical role in mammary gland development. ER is a member of the nuclear receptor superfamily, which comprises 48 proteins (1) that have a diversity of roles and are major contributors to the functioning of the endocrine system. As a nuclear receptor, ER has a DNA-binding domain (DBD) that enables it to directly regulate gene expression events and a ligand-binding domain (LBD) that renders it responsive to an activating ligand, namely oestrogen. The role of ER in initiating timely and controlled cell division during mammary gland development and during post-pubertal physiological functions, such as pregnancy, is a co-ordinated process that involves other hormones and their nuclear receptor transcription factors, including progesterone and prolactin (2).

The ability of ER to associate with DNA and initiate gene transcription is subverted in disease, where ER becomes a driving transcription factor that is no longer regulated by control mechanisms, and this results in an oestrogen-induced tumour. Essentially, ER continues to operate in its normal role as a gene regulating transcription factor, but the ER-mediated cell division occurs in an uncontrolled manner, resulting in tumour initiation and cancer progression. Three quarters of all breast cancers (~37000 out of 50000 new cases in the UK per annum) (source: Cancer Research UK) are characterized by the presence of ER. These cancers are therefore defined as ER^+^ and these women as candidates for specific treatments that block ER activity. One of the first targeted agents in the treatment of cancer was the selective oestrogen receptor modulator (SERM) tamoxifen, which is an effective treatment for ER^+^ breast cancers (3) because it can mimic oestrogen and bind to the LBD pocket of the ER, but unlike oestrogen, it alters the structure and function of ER so that this transcription factor is no longer capable of regulating gene expression (4). It has been estimated that almost half a million women are alive today because of the use of tamoxifen in the treatment of ER^+^ breast cancer (5) and although tamoxifen has been the mainstay for the treatment of ER^+^ disease for numerous years, many women develop endocrine resistance and tamoxifen subsequently fails. This led to the development of novel agents that block ER function, resulting in pure steroidal antioestrogens, such as Fulvestrant (Faslodex) and a class of compounds termed aromatase inhibitors (AIs). Fulvestrant binds to the LBD of ER, but unlike tamoxifen, it induces degradation of the ER protein, and this drug has been an effective treatment in tamoxifen-resistant contexts (6). In pre-menopausal women, the major source of oestrogen is ovarian production, but in post-menopausal women, the bulk of the oestrogen is metabolized from chemical precursors by an enzyme called aromatase. AIs work by blocking this metabolic step, essentially starving the cancer of its ligand, oestrogen. These different classes of drugs inhibit ER function, but they take distinct routes, meaning that resistance to one type of drug does not necessarily render other classes of compounds redundant and as such, different endocrine agents are used sequentially for the treatment of ER^+^ breast cancer.

The majority of women with ER^+^ disease will benefit from targeted drugs that block the ER pathway, but one-third of women will develop drug resistance (7). Understanding the mechanisms of drug resistance is a long-standing question and it is clear that cancers can circumvent ER-blocking agents via a number of different mechanisms. During the process of drug resistance, the tumour continues to grow and metastasizes to a secondary organ, particularly the bone, liver, brain and lung, where survival is compromised. A small fraction of tumours (~10–20%) lose ER expression (8) and there is evolving evidence that additional nuclear receptors can substitute for ER in this situation. Specifically, androgen receptor (AR) is known to be expressed in 80–90% of ER^+^ breast cancers (9) and there is a recent evidence showing that in the absence of ER, AR can substitute for ER and initiate cell division in an ER-independent, but nuclear receptor-dependent manner (10, 11). The bulk of drug-resistant breast cancers retain the expression of ER (8) and this transcription factor complex gets re-engaged even in the presence of an endocrine agent that inhibits the ER pathway. There are a number of mechanisms a cancer cell can utilize to circumvent either an ER-blocking chemical (i.e. tamoxifen), low levels of ER (i.e. Fulvestrant) or low levels of oestrogen (i.e. AIs), and these include: 1) changes in the levels of associated proteins that are required for ER transcriptional activity, termed co-factors (these will be discussed later); 2) upregulation of growth factor pathways that can initiate or promote ER transcriptional activity via kinase signalling pathways that phosphorylate target proteins to render them more active; 3) changes in drug metabolism and cellular secretion and 4) changes in the fidelity of the key proteins involved in the ER complex. Excellent reviews on the mechanisms of endocrine resistance have been described in other studies (12, 13). Recent findings have shown that 18–55% of metastatic samples harbour mutations in ER (*ESR1*) and these mutations occur in predictable amino acid residues in the LBD of ER, decreasing the dependence on oestrogen and the response to targeted treatments (14, 15, 16). Given the highly fecund nature of cancer cells and the general genomic instability of these cells, it is unsurprising that mutations and genomic alterations accumulate at a regular rate in cancer, making the disease a constantly evolving, moving target.

### Mechanisms of ER association with DNA in breast cancer

Understanding how ER initiates tumour formation is of paramount importance since it is likely that the underlying mechanisms that govern ER tumour formation are altered during the transition to drug resistance and metastasis. Decades of research have revealed extraordinary insights into how ER functions, with a complex picture emerging. Many of the facets of this mechanism of ER transcriptional activity are retained and conserved with other nuclear receptors in cancer. This is particularly the case in prostate cancer, where AR is the driving nuclear receptor and consequently, substantial parallels exist between AR-mediated prostate cancer development in men and ER activity in breast cancer in women.

For many years, ER was thought to be a stand-alone transcription factor, which in response to oestrogen was able to directly interact with DNA. A well-established ER consensus DNA sequence is composed of two inverted sequences separated by three random nucleotides (GGTCAnnnTGACC) (17). Once on the DNA, it was purported that ER could initiate gene transcription, hence making the ligand (oestrogen) and the receptor (ER) the sole determinants of its activity. The discovery of ER-associated co-factors (18, 19, 20) and the subsequent characterization of these factors revealed extraordinary insight into the complexes that form with ER to permit transcriptional regulation. It is now clear that ER activity requires the co-ordinated accumulation of dozens of co-factors that perform a multitude of functions. These include the ability to ‘open’ chromatin, making the compacted DNA accessible for ER to bind, proteins that provide platforms for other essential factors and numerous co-factors that have enzymatic properties that are required for optimal protein assembly and activity. A number of reviews describe the different co-factors and their roles in ER^+^ breast cancer (21, 22). The levels of key co-factors can be altered such that ER transcriptional activity is pushed in a positive or negative way by changes in critical but rate-limiting co-factors (20, 23), and this has been a documented way of circumventing the anti-proliferative action of endocrine therapies.

The study of a small number of ER target genes revealed insight into how ER can interact with DNA and regulate transcription (24, 25), but the advent of genomic technologies provided the first opportunity to assess ER function in an unbiased manner. By purifying ER-associated DNA (i.e. the genomic binding sites) by a method called chromatin immunoprecipitation (ChIP) and identifying the associated DNA by tiling microarrays and subsequently by high-throughput DNA sequencing, unknown ER binding sites were identified from breast cancer cell line models (26, 27, 28, 29). ER was thought to associate with the promoters of target genes, but unbiased mapping approaches showed that ER typically associates with enhancer elements that can be at considerable distances from the putative target gene (26). Interrogation of the thousands of ER-DNA interaction sites uncovered novel ER-associated proteins, which also interact with DNA and contribute to stabilizing the ER complex on the chromatin. These factors were identified by the over-representation of their consensus DNA binding motifs within the regions bound by ER, implying a functional connection at the enhancer elements occupied by ER. These included a number of transcription factors that can assist in tethering ER to the DNA, including FOXA1, GATA3, PBX1 and AP2γ (26, 30, 31, 32). It is unclear if all of these proteins are required or what degree of redundancy exists between these factors (33), but when any one of these individual protein is specifically inhibited in breast cancer cells, ER–DNA interactions are perturbed. As such, they all contribute, to some degree, in creating or maintaining ER interactions with the chromatin.

Given that most (~95%) ER binding sites are not at promoter proximal regions and instead occur at distal enhancers (27), a challenge was to identify whether all ER binding events were active and which gene targets were regulated. An indicator of a transcriptionally active ER binding enhancer is the presence of associated co-factors, such as AIB1, p300 and CBP (25, 34, 35). The presence of these (and other) important co-factors demarcate a functional, transcriptionally active ER binding element and genome-wide mapping of these factors has revealed that a subset of the many tens of thousands of ER–DNA contact sites are transcriptionally active. Identifying what target genes are induced or repressed by a specific ER binding site (that is typically far from any coding gene) has been an additional challenge that has been approached by exploiting methods for identifying chromatin loops (36) that form between enhancers (ER binding sites) and promoters of putative target genes. Candidate-based approaches can be made to investigate specific chromatin interactions, such as an ER-binding domain and the closest oestrogen-regulated gene promoter (26, 37). Unbiased approaches have been developed, which provide a the global snapshot of the interactome that occurs between ER binding events and their target gene (38). To add complexity to this system, it is now clear that not only can the ER complex reach over significant distances to regulate coding genes, but the ER–DNA binding complex that associates with enhancer elements can also contribute to localized transcription of non-coding RNAs, including enhancer RNAs (eRNA) that are produced from the actual site of ER occupancy (39, 40). A surprisingly large proportion of the genome of a breast cancer cell line is transcribed in response to oestrogen stimulation, much of which become RNAs that are not translated to proteins, but potentially play functional roles. Defining what non-coding RNAs are important and what their potential roles are, is an important question for future research.

### FOXA1 and GATA3 in breast cancer

FOXA1 was discovered by the enrichment of Forkhead motifs within ER binding sites (26, 41). FOXA1 is termed a pioneer factor (42) since it has the ability to occupy compacted DNA without the requirement for any additional proteins (43, 44) and can subsequently facilitate interactions between additional factors (such as ER) and the DNA (26). FOXA1 was shown to be required for all ER binding sites in models of ER^+^ breast cancer, and was also shown to be required for ER binding and growth of endocrine-resistant breast cancer cell line models (45). Immunohistochemistry of ER and FOXA1 in metastatic tumour material showed that FOXA1 is expressed in almost all solid distant metastases, and that correlation between ER and FOXA1 protein expression is high (46). The dependence on FOXA1 for ER function, even in endocrine-resistant contexts (45) creates a novel opportunity for therapeutic intervention, whereby targeting the pioneer factor, namely FOXA1, instead of the nuclear receptor (ER) might provide an opportunity for blocking ER transcriptional activity. Acquisition of activating ESR1 mutations, changes in co-factors levels or upregulation of growth factor pathways can all enable ER to activate gene expression in the presence of drugs, but all are dependent on ER making contact with the DNA. Inhibition of FOXA1 would theoretically circumvent these mechanisms associated with drug resistance, by destabilising ER–chromatin interactions. However, transcription factors are notoriously difficult to drug and a more realistic option might be the identification and subsequent therapeutic manipulation of upstream regulatory enzymes that influence FOXA1 function. The related protein, FOXA2, is known to be phosphorylated by the AKT pathway, which influences its cellular localization and function (47), although it is known that FOXA1 is not regulated by AKT (47). A concerted effort in identifying and characterizing FOXA1 regulatory enzymes is of paramount importance, given the evolving information linking FOXA1 with ER activity and the opportunity to block ER via its critical and necessary pioneer factor.

The third protein in the triumvirate of the ER complex is GATA3. *ER*, *FOXA1* and *GATA3* are three of the defining signature genes consistently observed in ER^+^ breast cancers (48, 49), and all three proteins have been shown to be required for the establishment of an oestrogen-responsive ER complex (50). Insights into the architecture within the ER DNA-binding domain was revealed by fine resolution transcription factor mapping (51). This showed predictable spacing between the motifs for ER, FOXA1 and GATA3, suggesting that all three proteins must be able to associate with the adjacent pieces of DNA for them to co-operate and form an oestrogen-responsive complex that is capable of generating a stable DNA interaction. Specific inhibition of GATA3 in breast cancer cells pushes ER towards new DNA binding sites that are demarcated by FOXA1 (52), suggesting that GATA3 might function as a rheostat, dictating possible ER–FOXA1 interactions. Total loss of GATA3 in mice mammary glands results in tumour progression and GATA3 was proposed to be a critical protein influencing cellular differentiation and tumorigenesis (53, 54). Interestingly, FOXA1 was shown to be a downstream target of GATA3 in the murine mammary gland (53). GATA3 has been shown to be required for the morphogenesis of normal mammary glands (55), suggesting an important role in promoting cellular differentiation, inhibiting proliferation and contributing towards the development of functional mammary glands. In ER^+^ breast cancer cells, silencing of GATA3 inhibited proliferation (30), suggesting a dependence on GATA3 for maintained proliferation of ER^+^ cancer cells. These findings provide a complex picture of GATA3 function, whereby it mediates cellular differentiation in normal mammary gland, but becomes an essential component within the ER complex during tumour formation.

As discussed above, ESR1 (ER) is commonly mutated in the metastatic context, but it is rarely mutated in primary tumours within the breast. FOXA1 and GATA3 on the other hand are mutated in primary breast cancer (56, 57, 58). *GATA3* is one of the most frequently mutated genes in breast cancer, with 14% of ER^+^ cases harbouring *GATA3* mutations. Recent data have shown that tumours that enrich cells with *GATA3* mutations tend to be ductal cancers, whereas tumours that possess *FOXA1* mutations tend to be of the lobular subtype (58). This distinction suggests that specific breast cancer subtypes are more tolerant of mutations in certain ER components and functionally do not benefit, or survive, from mutations in other components. It is currently unclear what the mutations in *FOXA1* and *GATA3* do to the function of these proteins and what effects these perturbations have on ER transcriptional activity. *FOXA1* mutations tend to occur in the DNA-binding domain (57), and preliminary data from prostate cancer suggest that mutations in FOXA1 decrease AR signalling and increase tumour growth (59). *GATA3* mutations can be roughly divided into two major classes: the first being within the second zinc finger DNA-binding domain of *GATA3* and the second class being C-terminal mutations that commonly induce frame shifts and an altered GATA3 protein (56). Whether both classes of *GATA3* mutations have the same effect on ER transcriptional activity and tumour outcome is currently unclear, but recent gene editing tools, such as CRISPR technologies, will permit investigation of these questions.

### Models of ER+ breast cancer and the complexity of hormonal crosstalk

The ER^+^ breast cancer research field is hampered by the scarcity of models. A limited number of ER^+^ PR^+^ cell lines that are responsive to endocrine drug treatment exists. The ‘workhorse’ in the field is the MCF-7 breast cancer cell line, which has helped to resolve a substantial amount of information around ER function. This included the discovery of the most robust oestrogen-regulated genes (60, 61, 62), which are validated as important signature genes in primary tumours (63, 64, 65). The co-factors and associated transcription factors discovered in these cell line models are critical factors in primary tumours, and the properties associated with ER binding events from this cell line model accurately reflect the observations made from ER ChIP-seq experiments carried out in primary tumour samples (46). More complex models representing ER^+^ cancer are becoming available, in the form of patient-derived xenograft (PDX) tumours, which provide numerous additional models for the investigation of ER^+^ disease, although the primary tumours that typically engraft in mice, such as PDX tumours, tend to be the more aggressive ER^+^ cancers (66). That said, the advent of PDX models has created an outstanding opportunity for discovering the mechanisms that contribute to drug resistance and for evaluating novel agents using *in vivo* systems that better represent ER^+^ disease.

**Figure 1:**
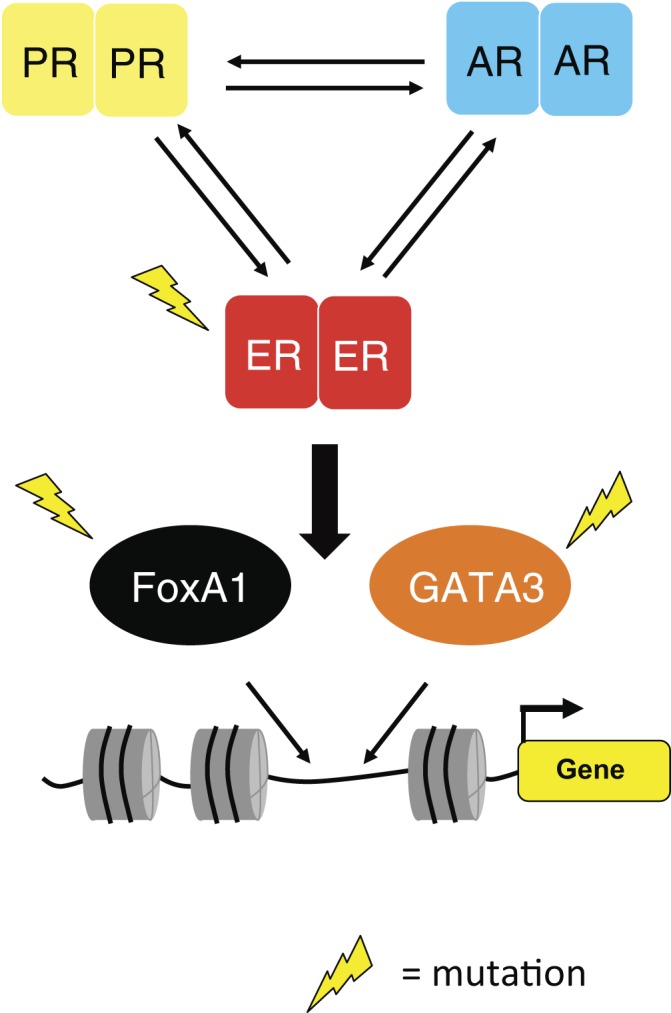
Oestrogen receptor (ER) uses pioneer factors to associate with DNA. Two critical proteins involved in tethering ER to the DNA include FOXA1 and GATA3. Both FOXA1 and GATA3 are mutated in primary cancers, whereas ER is mutated in metastases. The impact that these mutations have on ER activity is not known. Recently, the crosstalk between different nuclear receptors has become apparent. Progesterone receptor (PR) and androgen receptor (AR) are commonly expressed in ER^+^ breast cancer and both are known to impinge on ER transcriptional activity. A major challenge involves identifying how we can exploit existing PR and AR ligands for therapeutic use and how the mutations in *ER*, *FOXA1* and GATA3 influence this hormonal crosstalk.

The use of cell line models has permitted the ability to ‘strip’ out all hormones from the growth media, in order to study a single hormone and the downstream consequences. This has proven to be useful for studying a specific nuclear receptor and almost all the literature characterizing ER function, or PR function in breast cancer models have been conducted in the presence of oestrogen alone or progesterone alone, respectively. However, ER^+^ breast cancer is exposed to a complex milieu of hormones, growth factors and other stimuli, and the study of a single-nuclear receptor, such as ER, in the absence of all hormones other than its cognate ligand (oestrogen) does not accurately reflect the physiological situations. Recent findings have shown a substantial degree of nuclear receptor crosstalk in ER^+^ breast cancer, with both PR and AR converging on the ER pathway (9, 67, 68, 69, 70). The impact of AR or PR in ER transcriptional activity can occur in a number of different ways, including direct alteration of ER–DNA interactions by AR or PR (69, 70), through sequestration of rate-limiting co-factors or potentially through regulation of ER protein levels (Fig. 1). Similar observations have been made between glucocorticoid and ER (71), where GR is able to influence ER–DNA binding sites and subsequently the target genes that are regulated by the ER complex. The ability of nuclear receptors to interact within the same cellular environment is highlighted by the fact that different nuclear receptors can sometimes substitute for one another. A subtype of breast cancer called molecular apocrine, which comprise ~4% of all breast cancers, is characterized by gene expression signatures that are similar to ER^+^ subtypes (72, 73), but these cancers are ER negative. In this specific subtype of cancer, it is believed that AR can substitute for ER and can become the driving transcription factor, where it continues to regulate ER target genes because FOXA1 recruits AR (instead of ER) to the enhancers normally occupied by ER. Similarly, there is evidence that following AI treatment, a resistance mechanism involves down regulation of ER and subsequent mobilization of AR as the driving factor (10, 11). These findings provide the impetus to study ER in the presence of physiologically accurate hormonal conditions. The existence of hormonal crosstalk also reveals novel opportunities for therapeutic intervention, whereby parallel pathways are potentially drugged to indirectly regulate ER activity. Future work will identify who would gain the most benefit from PR or AR-targeted drugs for the treatment of specific ER^+^ breast cancer cases, a hypothesis that is supported by a wealth of clinical data showing that PR agonists have efficacy in breast cancer patients selected only based on ER^+^ status (74, 75, 76, 77, 78, 79, 80, 81, 82).

## Concluding remarks

The research community has studied the ER pathway for decades and the findings have revealed a complex picture, where ER associates with hundreds of proteins, interacts with thousands of regions in the genome and can regulate a multitude of target genes and non-coding RNAs, many of which are only now being identified. The findings from the study of this pathway have identified the mechanisms of drug resistance and novel ways of targeting this disease, which has translated to improved survival rates in women with this disease. The advent of immunotherapy in combination with existing and novel targeted agents is likely to improve the survival rates even more, but women with ER^+^ breast cancer continue to die and as such, the research community needs to continue the exploration of this important pathway. The use of better models (i.e. PDX) will contribute to this and, importantly, our ability and motivation to study drug resistance by analysing metastatic material and using models of metastasis is of paramount importance, as evidenced by the recent observation that ER itself is frequently mutated in metastases, something that was largely overlooked for decades. The substantial parallels between different hormonal cancers mean that insights generated from one system (such as breast cancer) will inform our understanding of other diseases (such as prostate and ovarian cancers), and the tools, technologies and biological observations need to be translated and exploited in diseases with common underlying pathological properties.

## Declaration of interest

I declare that I do not have any conflict of interest.

## Funding

Funding was received from Cancer Research UK and the funding code is ‘core funding’.
